# Supplementary material for: A fructose/H+ symporter controlled by a LacI-type regulator promotes survival of pandemic Vibrio cholerae in seawater
Source: Nat Commun. 2021 Jul 30;12:4649. doi: 10.1038/s41467-021-24971-3 (PMC8324912; doi:10.1038/s41467-021-24971-3)
Supplement: Supplementary file 1 — Supplementary Information [file 41467_2021_24971_MOESM1_ESM.pdf]

SUPPLEMENTARY INFORMATION

A fructose/H^+ symporter controlled by a LacI-type regulator promotes survival of pandemic *Vibrio cholerae* in seawater

Yutao Liu[1,2,3,6], Bin Liu[1,2,3,6], Tingting Xu[1,2,3,4,6], Qian Wang[1,2,3], Wendi Li[1,2,3], Jialin Wu[1,2,3], Xiaoyu Zheng[1,2,3], Bin Liu[1,2,3], Ruiying Liu[1,2,3], Xingmei Liu[1,2,3], Xi Guo[1,2,3], Lu Feng[1,2,3,*], Lei Wang[1,2,3,5,*]

[1] The Key Laboratory of Molecular Microbiology and Technology, Ministry of Education, Tianjin 300071, P. R. China;

[2] TEDA Institute of Biological Sciences and Biotechnology, Nankai University, TEDA, Tianjin 300457, P. R. China;

[3] Tianjin Key Laboratory of Microbial Functional Genomics, Tianjin 300457, P.R. China;

[4] Shenzhen Institute of Respiratory Diseases, Second Clinical Medical College (Shenzhen People's Hospital), Jinan University, Shenzhen 518020, P. R. China;

[5] State Key Laboratory of Medicinal Chemical Biology, Nankai University, Tianjin 300071, P. R. China

[6] Yutao Liu, Bin Liu, Tingting Xu contributed equally to this work.

[*] To whom correspondence may be addressed. E-mail: wanglei@nankai.edu.cn or fenglu63@nankai.edu.cn

This Supplementary Information includes:

Supplementary Figures 1 to 6

Supplementary Tables 1 to 2

Supplementary Figures

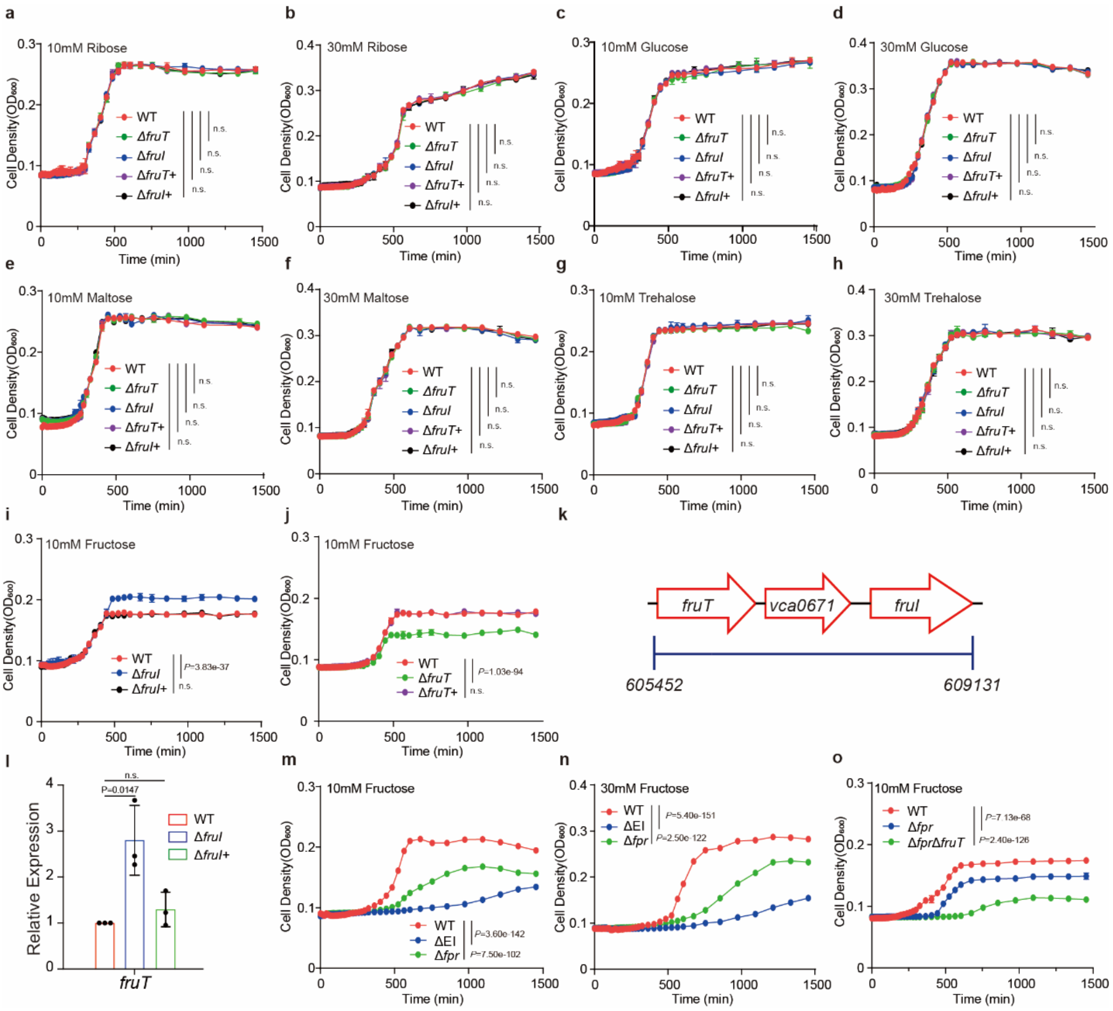

Supplementary Figure 1. FruI represses the expression of fructose transporter FruT, but is not involved in the regulation for the transport and utilization of other sugars. a-h, Growth curves of WT, Δ*fruI*, Δ*fruI*+, Δ*fruT* and Δ*fruT*+ in M9 medium containing 10 mM or 30 mM ribose (**a, b**), glucose (**c, d**), maltose (**e, f**) or trehalose (**g, h**) as the only carbon source. **i, j,** Growth curves of WT (**i, j**), Δ*fruI*(**i**), Δ*fruI*+(**i**), Δ*fruT*(**j**) and Δ*fruT*+(**j**) in M9 medium containing 10 mM fructose as the only carbon source. Data represent the mean ± SD (n = 3). **k,** Graphic representation of the region surrounding *fruI* in the genome of *V. cholerae* N16961 segment 605452–609131. Arrows represent open reading frames. **l,** qRT-PCR expression level of *fruT*

in WT, Δ*fruI* and Δ*fruI*+ in M9 medium containing 10 mM fructose as the only carbon source. Data represent the mean ± SD (n = 3). **m, n,** Growth curves of WT, ΔEI and Δ*fpr* in M9 medium containing 10 mM(**m**) or 30 mM(**n**) fructose as the only carbon source. Data represent the mean ± SD (n = 3). **o,** Growth curves of WT, Δ*fpr* and Δ*fpr*Δ*fruT* in M9 medium containing 10 mM fructose as the only carbon source. Data represent the mean ± SD (n = 3). Significance is determined by two-way ANOVA (**a-j, m-o**) or two-tailed unpaired Student's *t* test (**l**) and indicated as the P value; n.s. means no significant difference. Source data are included in Source Data file.

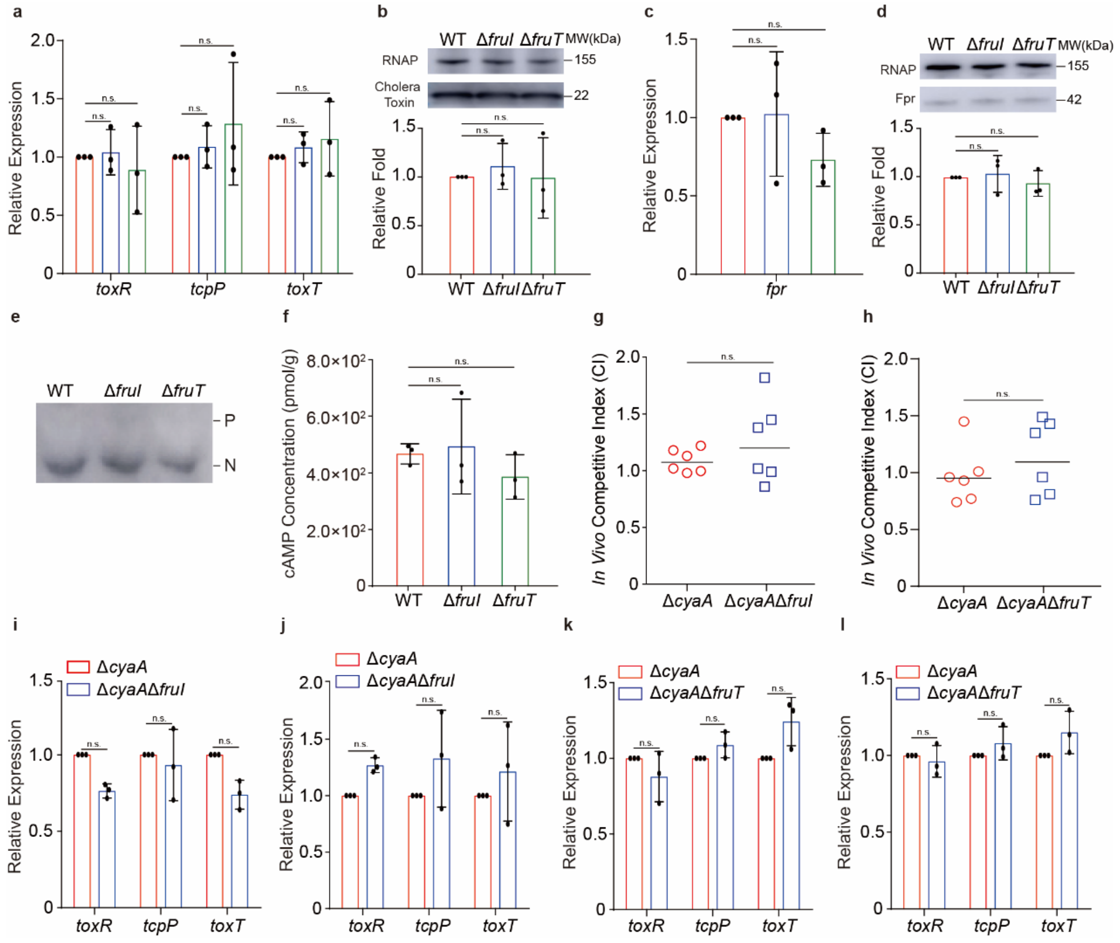

Supplementary Figure 2. FruI contributes to the virulence of *V. cholerae* by regulating the intracellular cAMP level. **a,** qRT-PCR expression level of virulence genes in WT, Δ*fruT* and Δ*fruI* in M9 medium containing 20 mM glucose as the only carbon source. Data represent the mean ± SD (n = 3). **b,** Representative western blotting images and quantitative analysis of cholera toxin in WT, Δ*fruT* and Δ*fruI* in M9 medium containing 20 mM glucose as the only carbon source. RNA polymerase (RNAP) was used as a loading control. Data represent the mean ± SD (n = 3). **c,** qRT-PCR expression level of *fpr* in WT, Δ*fruI* and Δ*fruT* in M9 medium containing 20 mM glucose as the only carbon source. RNA polymerase (RNAP) was used as a loading control. Data represent the mean ± SD (n = 3). **d,** Representative western blotting images and quantitative analysis of Fpr in WT, Δ*fruT* and Δ*fruI* in M9

medium containing 20 mM glucose as the only carbon source. Data represent the mean ± SD (n = 3). **e,** Phosphorylation status analysis of Fpr in WT, Δ*fruI* and Δ*fruT* in M9 medium containing 20 mM glucose as the only carbon source. P: phosphorylated protein; N: non-phosphorylated protein. **f,** cAMP concentrations in WT, Δ*fruI* and Δ*fruT* in M9 medium containing 20 mM glucose as the only carbon source. Data represent the mean ± SD (n = 3). **g, h,** Competition assay comparing the colonizing ability of Δ*cyaA*, Δ*fruI*Δ*cyaA*(**g**) and Δ*fruT*Δ*cyaA*(**h**) in infant mouse intestine (n = 6 mice per group). CI is defined as the output ratio of Δ*cyaA* or double mutants to Δ*cyaA lacZ-* divided by the input ratio of Δ*cyaA* or double mutants to Δ*cyaA lacZ-*. Each symbol represents the CI in an individual mouse; horizontal bars indicate the median. **i-l,** qRT-PCR expression level of virulence genes in the Δ*cyaA*, Δ*fruI*Δ*cyaA*(**i, j**) and Δ*fruT*Δ*cyaA*(**k, l**) in M9 medium supplemented with 20 mM fructose (**i, k**) as the only carbon source or in mouse small intestine (**j, l**). Data represent the mean ± SD (n = 3). Images are representative of three independent experiments (**e**). Significance is determined by two-tailed unpaired Student's *t* test (**a-d, f, i-l, j-m**) or two-sided Mann-Whitney U test **(g, h**) and indicated as the P value; n.s. means no significant difference. Source data are included in Source Data file.

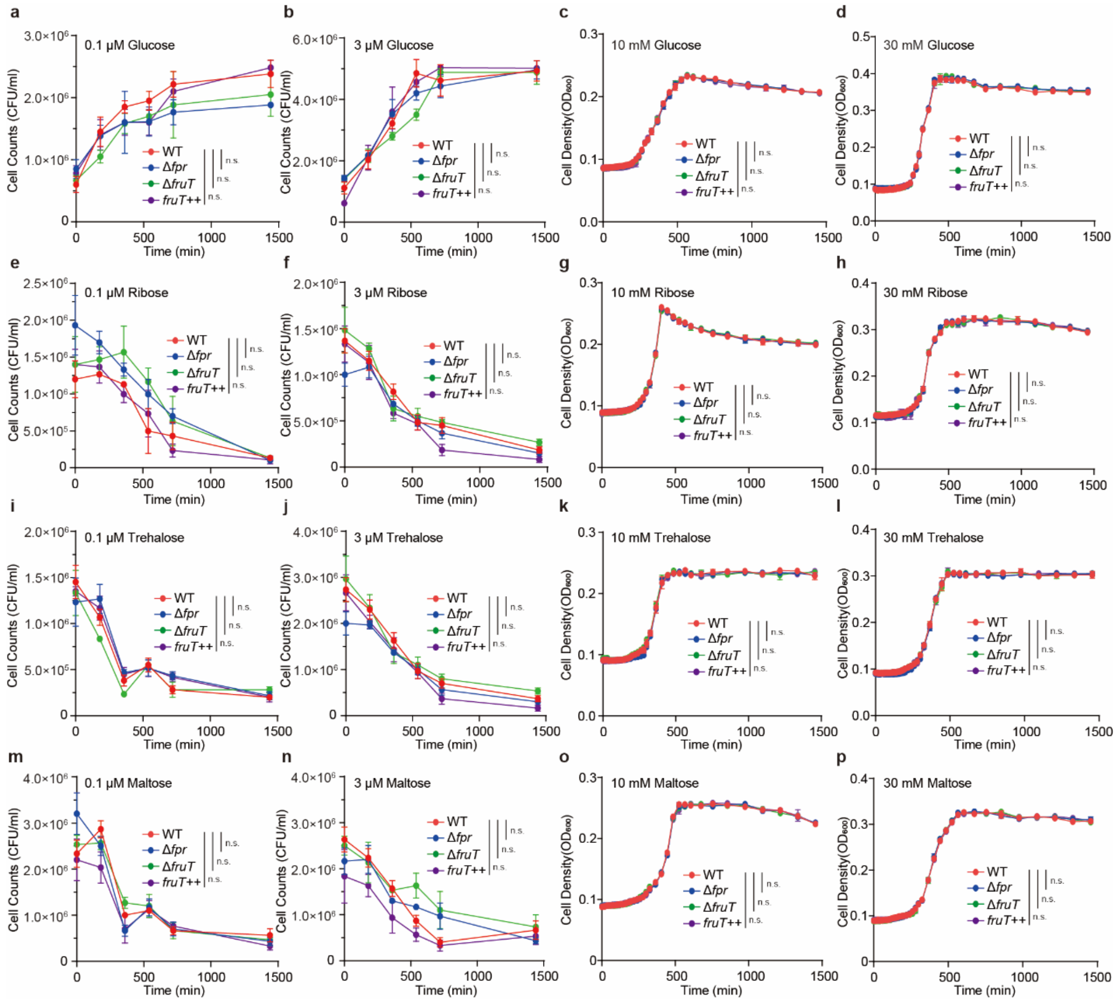

Supplementary Figure 3. FruT does not contributes to the uptake of sugars other than fructose. a-p, Growth curves of WT, Δ*fruT*, Δ*fpr* and *fruT*++ in M9 medium containing 0.1 μM, 3 μM, 10 mM or 30 mM glucose (**a-d**), ribose (**e-h**), trehalose (**f-l**) or maltose (**m-p**) as the only carbon source under aerobic conditions. Cell growth was followed by plating and determining the cell counts on LB agar plates (**a, b, e, f, I, j, m, n**) or measuring the absorbance at 600 nm (**c, d, g, h, k, l, o, p**). Data represent the mean ± SD (n = 3). Significance is determined by two-way ANOVA. Significance is indicated as the P value; n.s. means no significant difference. Source data are included in Source Data file.

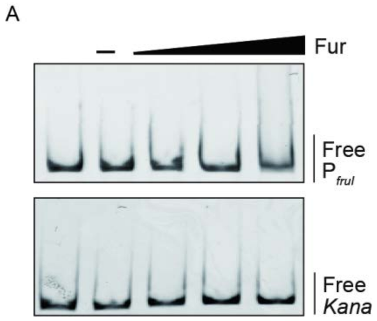

Supplementary Figure 4. EMSA of the binding of purified Fur protein to the promoter region of *fruI* and *kana* (negative control). Images are representative of three independent experiments. Source data are included in Source Data file.

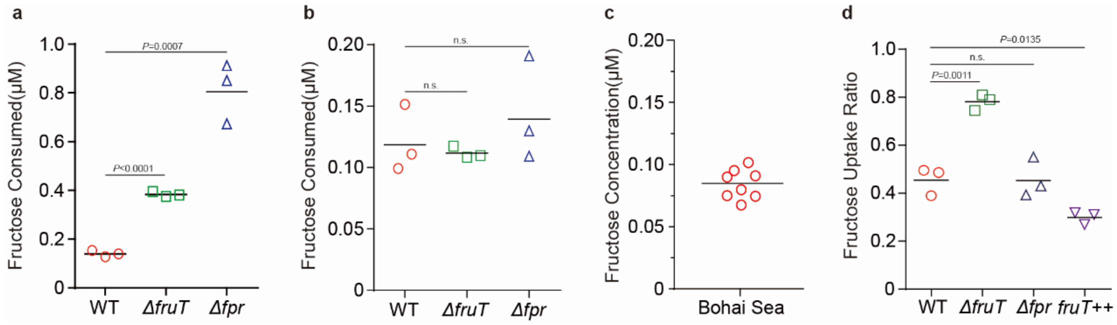

Supplementary Figure 5. FruT contributes to the fructose uptake of *V. cholerae* in seawater. a, b, Fructose concentration of WT, Δ*fruT* and Δ*fpr* growing in M9 supplemented with 30 mM(**a**) and 10 mM(**b**) fructose after 24 h (n = 3 sample per group). **c,** The fructose concentration in Bohai sea (n = 8 sample per group). **d,** Fructose uptake ratio in seawater samples after incubation with WT, Δ*fruT*, Δ*fpr* and *fruT*++ for 12h in seawater (n = 3 sample per group). The ratio is defined as the amount of rest fructose in seawater divided by total amount of fructose in original seawater samples. Significance is determined by two-tailed unpaired Student's *t* test (**a, b, d**) and indicated as the P value; n.s. means no significant difference. Source data are included in Source Data file.

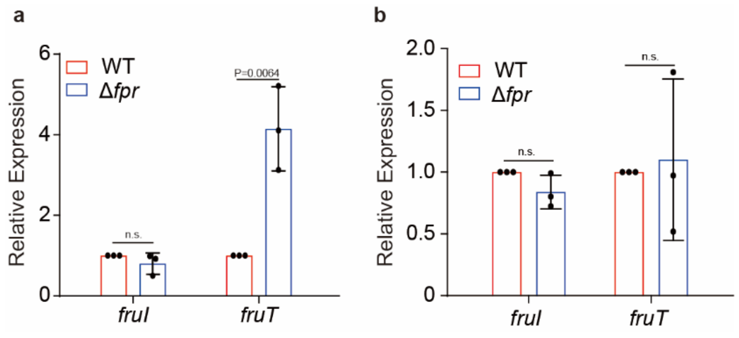

Supplementary Figure 6. Fructose PTS influences the expression of *fruT* via modulating bacterial fructose uptake. **a, b,** qRT-PCR expression level of *fruI* and *fruT* in WT and Δ*fpr* in M9 medium containing 20 mM fructose(**a**) or glucose(**b**) as the only carbon source. Data represent the mean ± SD (n = 3). Significance is determined by two-tailed unpaired Student's *t* test and indicated as the P value; n.s. means no significant difference. Source data are included in Source Data file.

Supplementary Tables

Supplementary Table 1. Strains and plasmids used in this study.

**Strains**	**Genotype or description**	**Source**
E12382	*Vibrio Cholerae* O1 El Tor strain E12382	Shanghai CDC*
S-17	*E. coli* S17-1/λpir strain	Lab collection
DH5α	*E. coli* DH5α/λpir strain	Lab collection
Δ*fruT*	*fruT* deletion mutant in E12382	This work
Δ*fruI*	*fruI* deletion mutant in E12382	This work
Δ*fpr*	*fpr* deletion mutant in E12382	This work
Δ*fruK*	*fruK* deletion mutant in E12382	This work
ΔEI	EI deletion mutant in E12382	This work
Δ*cyaA*	*cyaA* deletion mutant in E12382	This work
Δ*arcA*	*arcA* deletion mutant in E12382	This work
Δ*lacZ*	*lacZ* deletion mutant in E12382	This work
Δ*fruT*Δ*lacZ*	*fruT/lacZ* double deletion mutant E12382	This work
Δ*cyaA*Δ*lacZ*	*cyaA/lacZ* double deletion mutant E12382	This work
Δ*fruI*Δ*fruT*	*fruI/fruT* double deletion mutant in E12382	This work
Δ*arcA*Δ*fruT*	*arcA/fruT* double deletion mutant in E12382	This work
Δ*fruI*Δ*cyaA*	*fruI/cyaA* double deletion mutant in E12382	This work
Δ*fruT*Δ*cyaA*	*fruT/cyaA* double deletion mutant in E12382	This work
fruT++	E12382 containing pTrc99a-*fruT*	This work
WT (pBAD33)	E12382 containing pBAD33	This work
Δ*lacZ*(pBAD33)	Δ*lacZ* containing pBAD33	This work
Δ*fruT*+	Δ*fruT* containing pBAD33-*fruT*	This work
Δ*fruI*+	Δ*fruI* containing pBAD33-*fruI*	This work
Δ*fpr*+	Δ*fpr* containing pBAD33-*fpr*	This work
Δ*arcA*+	Δ*arcA* containing pBAD33-*arcA*	This work
HV55	BL21(DE3) containing pET28a-*fruI*	This work
HV63	BL21(DE3) containing pET28a-*arcA*	This work
HV64	BL21(DE3) containing pET28a-*fur*	This work
**Plasmids**		
pBAD33	Bacteria expression vector, CmR	Lab collection
pRE112	Suicide vector for mutation, CmR	Lab collection
pET28a	T7 expression vector, KmR	Lab collection
pKD4	Containing a kanamycin resistance cassette, KmR	Lab collection
pTrc99a	Bacteria expression vector, AmpR	Lab collection
pBAD33-*fruT*	pBAD33 carrying *fruT* from E12382， CmR	This work
pBAD33-*fruI*	pBAD33 carrying *fruI* from E12382， CmR	This work
pBAD33-*fpr*	pBAD33 carrying *fpr* from E12382， CmR	This work
pBAD33-*arcA*	pBAD33 carrying *arcA* from E12382， CmR	This work
pET28a-*fruI*	pET28a carrying *fruI* from E12382， KmR	This work

pET28a-*arcA*	pET28a carrying *arcA* from E12382，KmR	This work
pET28a-*fur*	pET28a carrying *fur* from E12382，KmR	This work
pTrc99a-*fruT*	pTrc99a carrying *fruT* from E12382，AmpR	This work

[*], Shanghai CDC, Shanghai Municipal Center for Disease Control & Prevention, China.

Supplementary Table 2. Primers used in this study (5'-3').

Primers for gene mutation		
fruI	P1	GCGGTACCAACGGCCATGTCTGCCGATTTGCA
fruI	P2	GGGGAGTGTCTTCCCCCCAACCGTTAGAACCGACTACAGCCTAGCAT
fruI	P3	ATGCTAGGCTGTAGTCGGTTCTAACGGTTGGGGGGAAGACACTCCCC
fruI	P4	TCCCCCGGGAGGGATGTCGGGATTGGATACGCT
fruT	P1	CGAGCTC ATACTCCTCTGGTTAACAAACCAG
fruT	P2	GAGTCATCCGCGCCTTGAATCAGGTGACCGATAAACCTCTGTTTTAAA
fruT	P3	ATTTAAAACAGAGGTTTATCGGTCACCTGATTCAAGGCGCGGATGACT
fruT	P4	CTAG TCTAGA CACAGTTAAACTCGGCATAACGAA
fpr	P1	CTAG TCTAGAGAAATACTCATCATCCAAAGCACG
fpr	P2	ACTTTTTTTGTCATGTGATGCCCCTCTTAACTCCTGTCTGCCTCTATA
fpr	P3	TATAGAGGCAGACAGGAGTTAAGAGGGGCATCACATGACAAAAAAA
fpr	P4	TCC CCCGGGTGCAGACAAGGCCGTTGCGAAACG
fruK	P1	CTAG TCTAGACATTTGGGCATTCTAAAGCAGCTG
fruK	P2	TTTCATCTTCACGACCTTCTGTCC GTGATGCCCCTTAACCTTCGCCTA
fruK	P3	TAGGCGAAGGTTAAGGGGCATCACGGACAGAAGGTCGTGAAGATGA
fruK	P4	TCC CCCGGG CACGACAGGCAGCATGTGCGATAC
cyaA	P1	CTAG TCTAGAACGAATACGGCTTTCGAGCTCAAG
cyaA	P2	TAGAAAACCTGAGACGTCAACGAGGTTTGCTTCCCTGATATGACAGA
cyaA	P3	ATCTGTCATATCAGGGAAGCAAACCTCGTTGACGTCTCAGGTTTTCTA
cyaA	P4	TCC CCCGGGGAGCTAAAGTATTCAATACCCCTA
arcA	P1	TCCCCCGGGCCAATCATAATCGATCAAGCATTG
arcA	P2	TCTAATTAGATATAAAAGAAGAGGTAGCGTAACCTAAACTTGTGAAA
arcA	P3	TTTCACAAGTTTAGGTAACGCTACCTCTTCTTTTATATCTAATTAGA
arcA	P4	GCTCTAGAGAAGATGACGCAACATCATGCCGG
lacZ	P1	CGGGATCCAGACCGGGAGCTAACGTA
lacZ	P2	GGCTTATTGTGGATCGGAGAAGTTGCGCATAAG
lacZ	P3	CCACAATAAGCCAGAGAGCCT
lacZ	P4	GTCTCGAGGATCGCTGTATTTCACTCG
EI	P1	CGAGCTCTAGTTCTAACACGAAAAGCCTGTG
EI	P2	ATCTCCTTGGTATCAACCGATAAAGCCTTACCTTAAACTGGTCAACT
EI	P3	AGTTGACCAGTTTAAGGTAAGGCTTTATCGGTTGATACCAAGGAGAT
EI	P4	GGGGTACCTTCTCTTCGAGCAGTGCCAAATCG
Primers for pBAD33 identifying		
pBAD33	F	ATGCCATAGCATTTTTATCC
pBAD33	R	GATTTAATCTGTATCAGG
pTrc99a	F	GAGCGGATAACAATTTCACACAGG
pTrc99a	R	GATTTAATCTGTATCAGG
Primers for complement construction		
fruI	F	CGAGCTCGAAAACAGCAAGGTTTGGATGT

fruI	R	GCTCTAGAGCCTAGGATTTGAGTGTACTTTTACGT
fruT	F	CGGAGCTCGATTTTTCCCGTTCTCTATC
fruT	R	GCTCTAGATTACGCGTTAGCCGGTTG
fpr	F	CGAGCTCGCGATTTTTGATCCTAGCCTAGT
fpr	R	GCTCTAGATTAACCTTCGCCTAAGCCAG
arcA	F	CGAGCTCACTCGTGATTGTTCTTAATATCTGTT
arcA	R	GCTCTAGATTAATCTTCTAAATCACCACCAGAAGCG
Primers for over-expression strain construction		
fruT	F	TCCCCCGGGATGACCACCAAAAAACACATGAGC
fruT	R	CGCGGATCCTTACGCGTTAGCCGGTTGGTTGCT
Primers for protein purification		
fruI	F	CGCGGATCCATGACTGATAAATCACGAGTTATG
fruI	R	CCGCTCGAGCTAGGATTTGAGTGTACTTTTACG
arcA	F	GGGAATTCCATATGATGCAAACCCCGCAGATCCTTATC
arcA	R	CCG CTCGAG TTAATCTTCTAAATCACCACAGAA
fur	F	GGGAATTCCATATGATGTCAGACAATAACCAAGCGCTA
fur	R	CCG CTCGAG TTATTTCTTCGGCTTGTGAGCGTT
Primers for EMSA		
P_{fruT}	F	TGATCACGGATCATTATCGC
P_{fruT}	R	GTAACCAACCAAGATCGCCA
P_{fruI}	F	GAGCTGTTTATTTTGGGTATAAC
P_{fruI}	R	CAGATACCGAGCTACCCGGTAC
kana	F	CATACGCTTGATCCGGCTAC
kana	R	CGGCCATTTTCCACCATGATAT
Primers for DNA pulldown		
P_{fruI}-biotin	F	GCTGCGATCTGGATTCACAC
P_{fruI}-biotin	R	TAGAACCGACTACAGCCTAGCA
fruI-biotin	F	GAGTCGTTGAAGGCACCAAA
fruI-biotin	R	CAGCAATCAGACGGCGTATG
Primers for qRT-PCR		
rrsA	F	ACCTTACCTACTCTTGACATCCA
rrsA	R	CCCAACATTTCACAACACGAG
fruT	F	AGGTTTGTCACTGGGCGGCTA
fruT	R	GCGGCAGCTAAAGGCGGAATT
fruI	F	ATACGCCGTCTGATTGCTGA
fruI	R	GTATGTCGTCTGGTATCCGAAGTG
tcpP	F	ATGGGGTATGTCCGCGTGAT
tcpP	R	TTTGGACAGGGGGCAGGATG
toxT	F	CGTTGGGCAGATATTTGTGGTG
toxT	R	CACTTGGTGCTACATTCATGGTTG
toxR	F	GCTCAAGCCGATAGAAGT
toxR	R	GATGCGTAAGGTTATGTTTT

fpr	F	GGTGCCATGTTAGTCGCCGAAG
fpr	R	CAGCATTGATCGCCACGCCTAG
