# Supplementary material for: A fructose/H+ symporter controlled by a LacI-type regulator promotes survival of pandemic Vibrio cholerae in seawater
Source: Nat Commun. 2021 Jul 30;12:4649. doi: 10.1038/s41467-021-24971-3 (PMC8324912; doi:10.1038/s41467-021-24971-3)
Supplement: Supplementary file 3 — Description of Additional Supplementary Files [file 41467_2021_24971_MOESM3_ESM.pdf]

Description of Additional Supplementary Files

File Name: Supplementary Data 1
Description: Distribution of 11 LacI-type regulator genes and *fruT* in pandemic and nonpandemic *V. cholerae* genomes.

File Name: Supplementary Data 2
Description: Differentially expressed genes between Δ*fruI* and WT in M9 medium containing 30 mM fructose. Differentially expressed genes in Δ*fruI* compared to WT were identified using edgeR (|fold change| > 2 and $P < 0.05$). P values were adjusted using the Benjamini and Hochberg approach to control the false discovery rate.
