# Supplementary material for: A fructose/H+ symporter controlled by a LacI-type regulator promotes survival of pandemic Vibrio cholerae in seawater
Source: Nat Commun. 2021 Jul 30;12:4649. doi: 10.1038/s41467-021-24971-3 (PMC8324912; doi:10.1038/s41467-021-24971-3)
Supplement: Supplementary file 6 — Reporting Summary [file 41467_2021_24971_MOESM6_ESM.pdf]

nature research

Corresponding author(s): Lei Wang

Last updated by author(s): Jul 5, 2021

Reporting Summary

Nature Research wishes to improve the reproducibility of the work that we publish. This form provides structure for consistency and transparency in reporting. For further information on Nature Research policies, see Authors & Referees and the Editorial Policy Checklist .

Statistics

For all statistical analyses, confirm that the following items are present in the figure legend, table legend, main text, or Methods section.

n/a	Confirmed	
☐	☒	The exact sample size (n) for each experimental group/condition, given as a discrete number and unit of measurement
☐	☒	A statement on whether measurements were taken from distinct samples or whether the same sample was measured repeatedly
☐	☒	The statistical test(s) used AND whether they are one- or two-sided<br>*Only common tests should be described solely by name; describe more complex techniques in the Methods section.*
☒	☐	A description of all covariates tested
☐	☒	A description of any assumptions or corrections, such as tests of normality and adjustment for multiple comparisons
☐	☒	A full description of the statistical parameters including central tendency (e.g. means) or other basic estimates (e.g. regression coefficient) AND variation (e.g. standard deviation) or associated estimates of uncertainty (e.g. confidence intervals)
☐	☒	For null hypothesis testing, the test statistic (e.g. F, t, r) with confidence intervals, effect sizes, degrees of freedom and P value noted<br>*Give P values as exact values whenever suitable.*
☒	☐	For Bayesian analysis, information on the choice of priors and Markov chain Monte Carlo settings
☐	☒	For hierarchical and complex designs, identification of the appropriate level for tests and full reporting of outcomes
☒	☐	Estimates of effect sizes (e.g. Cohen's d, Pearson's r), indicating how they were calculated

Our web collection on statistics for biologists contains articles on many of the points above.

Software and code

Policy information about availability of computer code

Data collection	RNA Sequencing data were collected by Illumina HiSeq 2500 (Illumina).
Data analysis	MedCalc (v12.3.0.0); Microsoft Excel 2019 (16.0.14026.20294); GraphPad Prism(v7.0.4); ImageJ (v1.8.0); edgeR; Peak Scanner (v1.0)

For manuscripts utilizing custom algorithms or software that are central to the research but not yet described in published literature, software must be made available to editors/reviewers. We strongly encourage code deposition in a community repository (e.g. GitHub). See the Nature Research guidelines for submitting code & software for further information.

Data

Policy information about availability of data

All manuscripts must include a data availability statement. This statement should provide the following information, where applicable:

- Accession codes, unique identifiers, or web links for publicly available datasets
- A list of figures that have associated raw data
- A description of any restrictions on data availability

All sequence data that support the findings of this study have been
deposited in the NCBI SRA database under the accession codes SRR12474047(https://www.ncbi.nlm.nih.gov/sra/?term=SRR12474047) and SRR12474048(https://www.ncbi.nlm.nih.gov/sra/?term=SRR12474048).
DNase I footprinting assay data have been deposited in OSF under accession code 4SQ62(https://osf.io/4sq62/).
Other source data are provided with this paper in the Source Data file.

Field-specific reporting

Please select the one below that is the best fit for your research. If you are not sure, read the appropriate sections before making your selection.

- [x] Life sciences
- [ ] Behavioural & social sciences
- [ ] Ecological, evolutionary & environmental sciences

For a reference copy of the document with all sections, see nature.com/documents/nr-reporting-summary-flat.pdf

Life sciences study design

All studies must disclose on these points even when the disclosure is negative.

Sample size	RNA-seq was performed once for the WT and ΔfruI sample. The pooled RNA collected from three different biological samples of the WT and ΔfruI were used for analysis. All other in vitro experiments were repeated at least three times ($n \geq 3$). Mice colonization experiments were conducted twice with at least 3 mice ($n \geq 3$) in each group, and the combined data for the two experiments was used for statistical analysis.
Data exclusions	No data were excluded from the analyses.
Replication	All the reported experiments were reproducible. Data reproducibility was confirmed by three independent experiments. RNA-seq results were validated by three independent qRT-PCR analyses on target genes.
Randomization	All samples were assigned to groups randomly.
Blinding	cDNA libraries were constructed and analyzed by NOVOGENE, Inc (TianJin, China), which also provided the statistical analysis. Other experiments were not done blindly. Most experiments were conducted by at least two different researchers who have not known the situation and results of the study in advance and repeated on at least two independent days.

Reporting for specific materials, systems and methods

We require information from authors about some types of materials, experimental systems and methods used in many studies. Here, indicate whether each material, system or method listed is relevant to your study. If you are not sure if a list item applies to your research, read the appropriate section before selecting a response.

Materials & experimental systems

n/a	Involved in the study	
☐	☒	Antibodies
☒	☐	Eukaryotic cell lines
☒	☐	Palaeontology
☐	☒	Animals and other organisms
☒	☐	Human research participants
☒	☐	Clinical data

Methods

n/a	Involved in the study	
☒	☐	ChIP-seq
☒	☐	Flow cytometry
☒	☐	MRI-based neuroimaging

Antibodies

Antibodies used	Anti-Fpr monoclonal antibody (custom-made by Willget Biotech Co., Ltd). 1:2,000 dilution used for immunoblotting.<br>Anti-RNA polymerase beta antibody cat.ab191598. Abcam. 1:2,000 dilution used for immunoblotting.<br>Anti-cholera toxin antibody cat.ab123129. Abcam. 1:2,000 dilution used for immunoblotting.<br>HRP-conjugated goat anti-rabbit IgG secondary antibody cat.EF0002. Sparkjade. 1:5,000 dilution used for immunoblotting.
Validation	Anti-Fpr monoclonal antibody was tested and validated by Willget Biotech Co., Ltd. Anti-RNA polymerase beta and Anti-cholera toxin were obtained commercially and were tested and validated by the respective company. All antibodies had validation statement provided on the website of the manufacturer.<br>Anti-Fpr antibody: rabbit monoclonal to Fpr; suitable for WB; Against peptide HGTTDTRELVKQT.<br>Anti-RNA polymerase beta antibody : rabbit monoclonal to RNA polymerase beta; Suitable for: IP, WB; Reacts with: Escherichia coli.<br>Anti-cholera toxin antibody: Rabbit polyclonal to Cholera Toxin; Suitable for: WB, ELISA; Reacts with: Other species.

Animals and other organisms

Policy information about studies involving animals; ARRIVE guidelines recommended for reporting animal research

Laboratory animals	Both sexes of CD-1 infant mice (5 days old)
Wild animals	This study did not involve wild animals.
Field-collected samples	This study did not involve samples collected from the field.
Ethics oversight	All animal studies were conducted according to protocols approved by the Institutional Animal Care Committee of Nankai University (Tianjin, China) and performed under protocol no. IACUC 2016030502.

Note that full information on the approval of the study protocol must also be provided in the manuscript.
